# Nutritional and lifestyle intervention strategies for metabolic syndrome in Southeast Asia: A scoping review of recent evidence

**DOI:** 10.1371/journal.pone.0257433

**Published:** 2021-09-14

**Authors:** Sze Mun Thor, Jun Wern Yau, Amutha Ramadas

**Affiliations:** Jeffrey Cheah School of Medicine and Health Sciences, Monash University Malaysia, Bandar Sunway, Malaysia; UCSI University, MALAYSIA

## Abstract

Metabolic syndrome (MetS) is frequently associated with various health issues and is a major contributor to morbidity and mortality worldwide, particularly with its recent relevance to coronavirus disease 2019 (COVID-19). To combat its increasing prevalence in Southeast Asia, numerous intervention programs have been implemented. We conducted a scoping review on recent interventions to manage MetS among Southeast Asians using standard methodologies. Cochrane, Embase, Ovid MEDLINE, PubMed, and Scopus databases were systematically searched to yield peer-reviewed articles published between 2010–2020. We included 13 articles describing 11 unique interventions in four Southeast Asian countries: Malaysia, Thailand, Indonesia, and Vietnam. These interventions were broadly categorized into four groups: (i) nutrition (*n* = 4); (ii) physical activity (*n* = 2); (iii) nutrition and physical activity (*n* = 2); and (iv) multi-intervention (*n* = 3). Most studies investigated the effects of an intervention on components of MetS, which are anthropometry, blood pressure, glucose-related parameters, and lipid profile. Significant improvements ranged from 50% of studies reporting serum triglyceride and HDL-cholesterol levels to 100% for waist circumference. Evidence on interventions for individuals with MetS remains limited in Southeast Asia. More studies from other countries in this region are needed, especially on the effects of dietary interventions, to effectively address gaps in knowledge and provide sufficient data to design the ideal intervention for Southeast Asian populations.

## Introduction

Metabolic syndrome (MetS) is an energy utilization and storage disorder characterized by a constellation of aberrant anthropometric and biochemical components. MetS is also known as Insulin Resistance Syndrome due to its apparent underlying pathogenetic factors of insulin resistance and abnormal fat deposition [1, 2]. Featuring elevated blood pressure and glucose levels, dyslipidemia, and obesity, MetS is conceptualized as a significant risk factor for cardiovascular diseases [3]. MetS also has been suggested to be associated with other deleterious health conditions, including erectile dysfunction, polycystic ovarian syndrome, colorectal cancer, and mental health disorders [3].

The definition of MetS has evolved since its introduction by Reaven as Syndrome X in the 1988 Banting Lecture [4]. In 1998, the World Health Organization (WHO) proposed that MetS should be defined by the presence of insulin resistance and two other abnormal parameters, including microalbuminuria [5]. The European Group for the Study of Insulin Resistance (EGIR) shared the sentiment, requiring non-diabetic individuals to have insulin levels in the 75^th^ percentile or higher, but excluded the microalbuminuria criterion [6]. Insulin resistance was also a prerequisite for MetS diagnosis according to the 2003 American Association of Clinical Endocrinology (AACE) criteria [7].

However, a different approach was taken by the National Cholesterol Education Program Adult Treatment Panel III (NCEP ATP III), in which insulin resistance was not mandatory for the diagnosis of MetS [8]. Several modifications to the NCEP ATP III definition were introduced in 2005, such as lowering the threshold for fasting glucose and optimizing waist circumference for Asians in the United States [9]. This was essentially performed to adequately account for individuals of Asian descent with marginally increased waist circumference not meeting the original diagnostic criterion. These individuals demonstrate apparent susceptibility to MetS, hence lowering the waist circumference threshold to ≥94 cm in men or ≥80 cm in women was warranted [9]. According to specified ethnicity values, the International Diabetes Federation (IDF) took it a step further by necessitating central obesity as measured by waist circumference [10]. As each preceding set of criteria carries slight differences which complicate MetS diagnosis, the Harmonized criteria were proposed in a joint interim statement by six international organizations in 2009 [11]. To best account for cardiovascular risk factors, the Harmonized criteria treat all components equally, with no single component identified as a prerequisite [11]. Currently, the NCEP ATP III, IDF, and modified NCEP ATP III criteria are most accepted and widely used in epidemiological surveys and national statistics [12].

Predominantly arising from sedentary behavior and excessive consumption of high-calorie foods, MetS affects many populations across the globe particularly where there is widespread adoption of Westernized lifestyles associated with improved travel convenience, sedentary leisure activities and increased dependency on fast foods [13]. Many elements predispose individuals to MetS, ranging from upstream factors such as socioeconomic status and residence to individual aspects, including genetics and lifestyle choices [3, 14]. MetS makes up a substantial proportion of non-communicable diseases worldwide and is a significant health and economic burden to developing and developed nations alike [13]. Failure to adequately address this pressing issue would translate to more significant morbidity and mortality due to cardiovascular disease, which is predicted to cause the deaths of 22.2 million people in 2030 [15]. Additionally, the recent coronavirus disease 2019 (COVID-19) pandemic has underscored pressing concerns regarding MetS and its associated conditions, which have been strongly linked to increased infection risk, higher severity of COVID-19 and worse prognosis [16].

Situated geographically between east India and south China, Southeast Asia is represented by the Association of Southeast Asian Nations (ASEAN): Singapore, Thailand, Malaysia, Indonesia, the Philippines, Laos, Vietnam, Cambodia, Myanmar, and Brunei. The 2020 estimates by the United Nations placed Southeast Asia as the third most populous subregion in the world, with a total population approaching 670 million [17]. This ethnically diverse region experienced unprecedented growth and development over recent decades, transforming many traditionally agricultural areas into bustling cities and revolutionizing jobs from farming and fishing to office-based work. While these changes bring prosperity to many, they act as catalysts to the rise of MetS and its components [18]. According to statistics by the WHO, a higher percentage of the population of most Southeast Asian countries is afflicted with raised blood glucose and blood pressure than that of the United States or the United Kingdom [16]. Furthermore, excluding Singapore, citizens of all nations in the region have a considerably higher probability of premature death due to non-communicable diseases, chiefly cardiovascular diseases such as stroke and coronary artery disease [16].

Findings from prevalence studies emphasized that MetS increases at a particularly alarming rate in this region [13, 19]. According to national survey data, MetS is highly prevalent in Malaysia and Indonesia. The former ranked the highest among selected major countries in the Asia Pacific region, with 37.1% of its adult population affected in 2008 [13]. By contrast, the lowest prevalence of MetS in the same region was found in the Philippines, which was 11.9% in 2003 [13]. Although the distribution of MetS is not uniform throughout Southeast Asia, evidence has shown that its prevalence continues to rise consistently over the past decades [19].

Therefore, effective strategies to combat MetS are imperative in reducing its impact on individuals, communities, and nations. Many interventions have been implemented in Western countries with varying degrees of success [20, 21], but little is known about their application in Southeast Asia. Hence, this scoping review describes interventions to manage MetS in Southeast Asia and their relative efficacy.

## Methods

### Study design

We utilized a scoping review design to comprehensively describe strategies for MetS employed throughout Southeast Asia and determine pertinent knowledge gaps in this critical research area. This scoping review was guided using a combinatory approach based on methodological frameworks by Arksey and O’Malley [22], the Joanna Briggs Institute [23], and Levac et al. [24], as well as the checklist developed by the Preferred Reporting Items for Systematic Reviews and Meta-Analyses Extension for Scoping Reviews (PRISMA-ScR) [25] (S1 Checklist). Ethical approval was not sought as we have only used published data for this review.

The operationalized definition for interventions in this review is any program or strategy that reduces the occurrence of MetS or its components within the study population. Since there is no commonly accepted definition for MetS, we accepted articles describing MetS using any known definitions [5–11] outlined in S1 Table. To improve the inclusivity of the research in MetS, we also included studies that recruited MetS individuals identified using other criteria as described by respective study researchers. We included studies performed among the adult population (aged 18 and above) in Southeast Asia with no restrictions on sample size, trial design, and intervention length, and provider.

### Data sources and search strategy

A literature search was conducted in five online databases: Cochrane, Embase, Ovid MEDLINE, PubMed, and Scopus. The search strategy consisted of the keyword ’metabolic syndrome’ combined with the Boolean operator AND with the countries of Southeast Asia and their populations. These are (’Southeast Asia’ OR ’Southeast Asian’), (’Malaysia’ OR ’Malaysian’), (’Singapore’ OR ’Singaporean’), (’Thailand’ OR ’Thai’), (’Myanmar’ OR ’Burma’ OR ’Burmese’), (’Cambodia’ OR ’Cambodian’), (’Laos’ OR Laotian’), (’Vietnam’ OR ’Vietnamese’), (’Brunei’ OR Bruneian’), (’Philippines’ OR ’Filipino) and (’Indonesia’ OR ’Indonesian’), linked with the Boolean operator OR.

Where possible, searches were limited to the adult population, the English language, and articles published between 1 January 2010 and 31 December 2020. This timeframe was selected to ensure that recent evidence was captured. S2 Table demonstrates the search syntax employed for Ovid MEDLINE, which was similarly applied to the other four electronic databases.

### Study selection

We utilized the Covidence software [26] to select studies for inclusion systematically. After importing all searched articles, duplicates were removed, and the articles were screened first by their titles and abstracts, followed by full texts according to the eligibility criteria. Manual hand-searching of reference lists of included studies was also performed to seek articles not identified in the database searches. Two independent researchers (T.S.M. and Y.J.W.) performed the study selection process with input from a third researcher (A.R.), who also arbitrated any disagreement. The final selection of articles was agreed upon by all three researchers and described interventions performed on any adult population in Southeast Asia, at least 80% of whom were required to have MetS. The screening of articles at both stages was conducted according to pre-determined study eligibility criteria (S3 Table).

### Data extraction

After the included articles were finalized, relevant data was systematically extracted in line with several descriptive variables—year, study design, subjects and sample size, country, criteria used to define MetS, intervention focus and duration, outcomes measured, and primary findings. The studies were subsequently classified according to the focus and outcomes of each intervention to establish this review’s key discussion areas.

## Results

### Selection of articles

**Fig 1:**
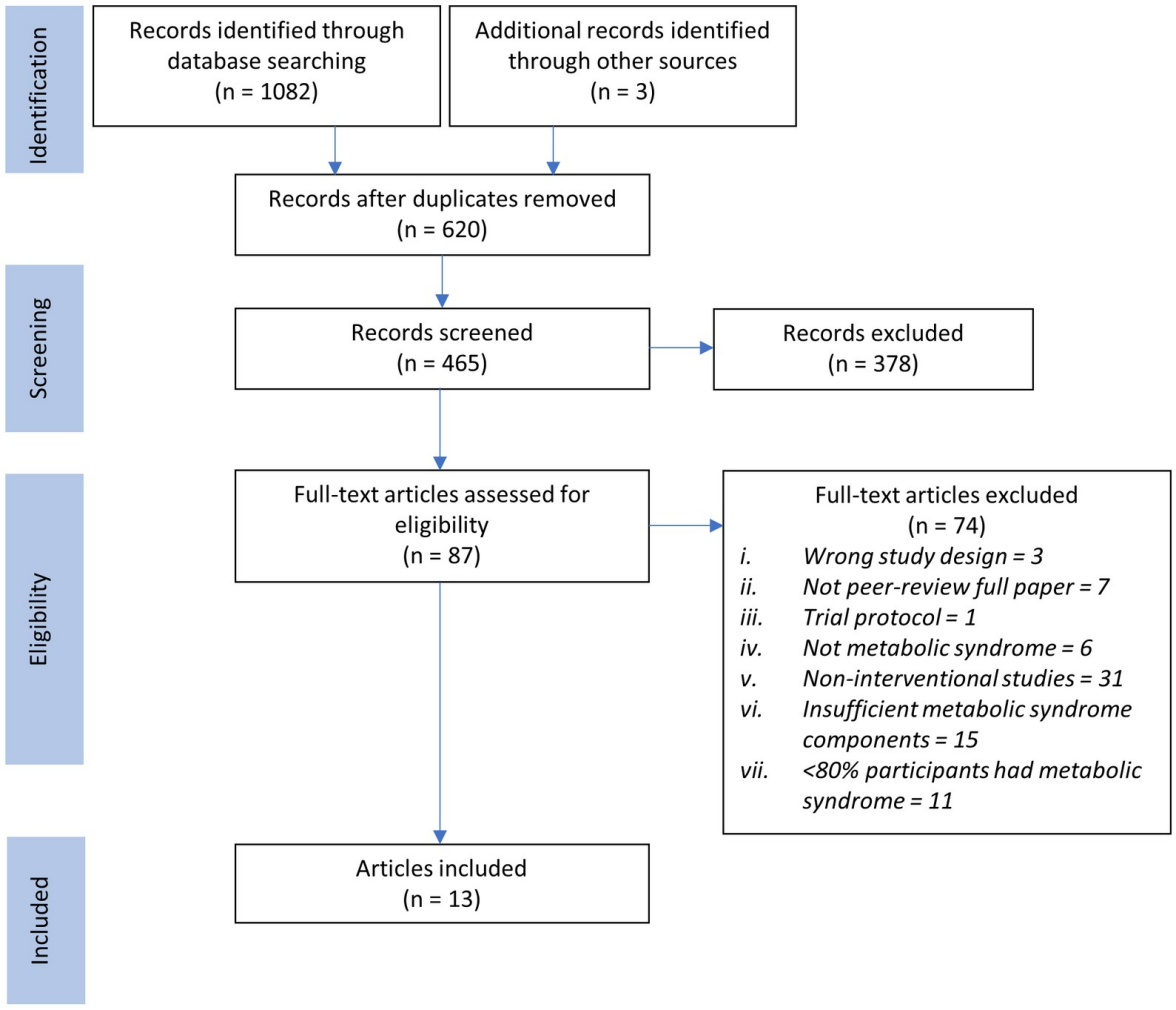
PRISMA 2020 flow chart delineating the study selection process.

Database searching resulted in 1082 records, while an additional three were retrieved by manual hand-searching. After removing 620 duplicates, 465 articles were subjected to screening by title and abstract, of which 378 were excluded mainly due to incorrect study designs, being trial protocols, and involvement of non-Southeast Asian populations. A full-text review was performed for the remaining 87 articles to yield the final total of 13 articles for inclusion. We found that the two articles by Tran et al. constitute the same trial but reported different outcomes; hence we attributed both as a single entry in our analysis [27, 28]. A similar method was used for Mahadzir et al.’s two articles [29, 30]. Fig 1 depicts the literature search process together with the selection of studies and rationale for exclusion.

### Study characteristics

**Fig 2:**
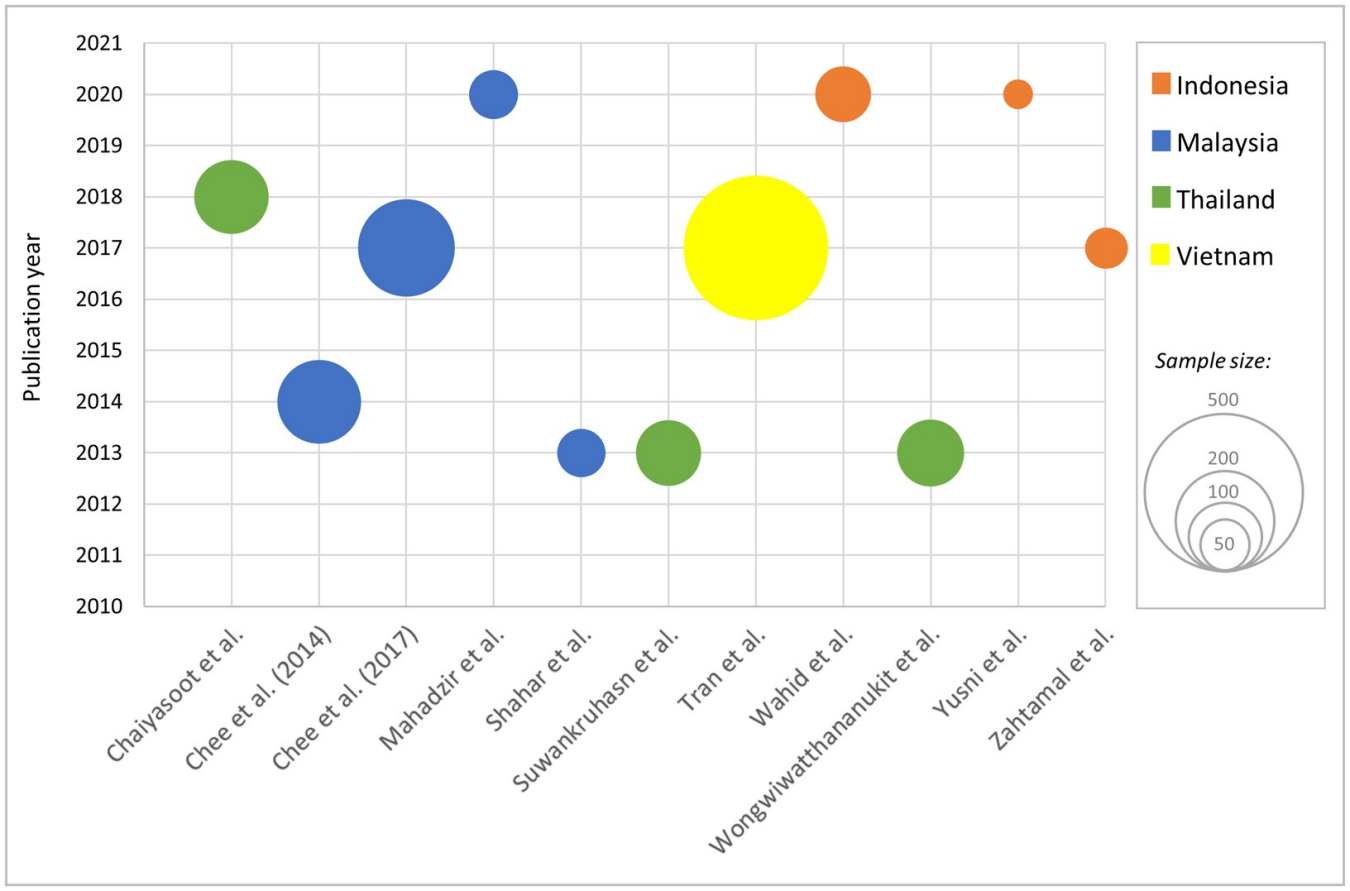
Bubble chart of each included study, its respective publication year and relative sample size.

Detailed information on each study can be found in greater detail in S4 Table. A total of 1,241 participants of 11 interventions conducted in four countries (Indonesia, Malaysia, Thailand and Vietnam) were included. Malaysians were most studied with 424 (34.2%) participants. This was followed by Vietnam with 417 (33.6%) participants even though only one trial was performed [27, 28]. All included studies were randomized controlled trials (RCTs) except for four, three of which were quasi-experiments [31–33], whereas one study was a feasibility trial with a pre-post design [30]. Analysis by publication year reveals studies were published in 2013, 2014, 2017, 2018, and 2020, while other years within our specified timeframe were devoid of relevant publication (Fig 2).

The Harmonized criteria was most used to define MetS among participants (*n* = 4), followed closely by the modified NCEP ATP III and IDF criteria with three and two studies, respectively. The study by Wahid et al. [34] has reported MetS criteria other than the known definitions, while another study by Yusni et al. [31] did not specify the criteria used.

### Intervention focus

Interventions for populations with MetS can be broadly classified into four groups targeting different aspects: (i) nutrition (*n* = 4); (ii) physical activity (*n* = 2); (iii) nutrition and physical activity (*n* = 2); and (iv) multi-intervention (*n* = 3). The majority of the studies were conducted among adults with MetS (*n* = 7) and within a community (*n* = 4). The most common intervention duration was 8–12 weeks (*n* = 5), followed by 16–25 weeks (*n* = 4). The majority of the interventions were delivered by a nutritionist or dietitian (*n* = 5). Table 1 provides an overview of the interventions.

**Table 1:** Summary of intervention characteristics of the included studies (*n* = 11).

Characteristics		*n* (%)
Intervention focus	Nutrition / nutraceuticals	4 (36.4)
	Physical activity	2 (18.2)
	Nutrition and physical activity	2 (18.2)
	Multi-intervention	3 (27.3)
Study participants	Young adults (18–40 years)	2 (18.2)
	Middle-aged adults (40–60 years)	5 (45.5)
	Older adults (>60 years)	4 (36.4)
Study setting	Community	4 (36.4)
	Community clinic / primary care	2 (18.2)
	Workplace	3 (27.3)
	Unknown	2 (18.2)
Duration of Intervention (weeks)	<4	2 (18.2)
	8 to 12	5 (45.5)
	16 to 25	4 (36.4)
Primary intervention provider	Nutritionist / dietitian	5 (45.5)
	Public health expert/physician	2 (18.2)
	Nurse	1 (9.1)
	Not known	3 (27.3)

#### Nutrition

Four studies [31, 33–35] reported in Southeast Asia focused on nutritional interventions within the recent ten years. Of the four, three studies [31, 34, 35] reported nutraceuticals or supplements.

Two studies reported on plant-based nutritional interventions: one on black seed oil [34] and the other on rosella (*Hibiscus sabdariffa L*.) tea [31]. Both studies were conducted in Indonesia and lasted for approximately three weeks. In the first study, 62 participants were allocated equally into two groups: a control group and an intervention group where 3mL of black seed oil—a *Nigella sativa* seed extract—was given daily for 20 days [34]. On the other hand, the quasi-experimental study on rosella tea was performed on 18 older women over 60 on treatment for hypertension and diabetes [31]. Twice a day, subjects in the intervention arm were given 2 grams (equivalent to 5 calyces) of rosella mixed with 150 ml of boiling water [31]. Concurrently, the control group continued with their antihypertensive and antidiabetic therapy without rosella.

Vitamin D supplementation was investigated by Wongwiwatthananukit et al. [35]. In this RCT, vitamin D_2_, also known as ergocalciferol, was given at two weekly doses of 40,000 IU and 20,000 IU for eight weeks to two groups of adult MetS patients with 25-hydroxyvitamin D deficiency [serum 25(OH)D levels of ≤20 ng/ml] [35]. Comparisons were made between the two interventional groups and a matched placebo group to evaluate 25(OH)D levels, metabolic syndrome components, and safety [35].

Interestingly, only one study reported on nutrition education in people with MetS [33]. In this quasi-experiment, 47 older Malays with MetS from rural Malaysia were recruited for a six-month education program. While the control group was provided a general health education package, the intervention group received four nutrition education sessions based on specifically developed packages. These sessions, comprised of talks, demonstrations of healthy cooking and exercise, and group counseling led by dietitians and nutritionists, aimed to instill good dietary practices to achieve healthy aging [33].

#### Physical activity

Interventions aimed to improve physical activity levels were explored in two RCTs conducted among Malaysian government employees [36, 37]. Both trials have intervention lengths of approximately four months and similar inclusion criteria: men and women 18–59 years old with MetS according to the Harmonized criteria at Stages 1–3 of the Stages of Change model [38] for physical activity behavior.

The 2014 trial involved 140 participants in a Facebook-based intervention involving posts and dialogue promoting physical activity [36]. On the other hand, the 2017 study recruited 189 participants for a three-arm trial, which were a point-of-decision prompt group comprising colored standing banners placed at strategic locations to provide motivation for increasing physical activity, an aerobics group consisting of weekly hour-long aerobics classes taught by a certified instructor featuring moderate-intensity exercise, and a control group. All three groups had group meetings every two weeks to monitor step-count progress [37].

#### Nutrition and physical activity

Lifestyle education involving both elements was the central interventional theme for two studies [27, 28, 39]. In the study involving the largest sample size in this review, Tran et al. investigated the effect of a combined physical activity and nutrition intervention on MetS components [27] and behavioral outcomes, including physical activity levels and dietary practices [28]. Physicians and nurses delivered the six-month community-based intervention. It consisted of four education sessions, distribution of resistance bands and information booklets containing tips on proper exercise techniques and healthy eating, and involvement in walking groups. On the other hand, the control group received standard advice on diet and physical activity. Importantly, this RCT is the only study in this review that assessed the proportion of participants with MetS before and after intervention [27].

Chaiyasoot et al. took a slightly different approach by conducting a 12-week dietitian-led lifestyle education to raise awareness of MetS-related health complications and achieve weight loss by improving nutritional and lifestyle behaviors [39]. This was delivered via a group session at baseline followed by one-on-one counseling sessions every fortnight. Subjects were assigned to two study groups, with one group given additional high-protein meals to replace two main meals every day [39].

#### Multi-intervention

In a quasi-experiment by Zahtamal et al., 34 employees from two companies in the oil refinery and plantation industries were involved [32]. A multilevel intervention was carried out consisting of health education lectures, printed materials and social support for workers, personal counseling for the employees’ families, advocacy for company leaders, and collaborative learning for all participants. The effects of these Working Health Promotion activities were compared with a control, in which workers received health education lectures only [32].

Two programs to support individual endeavors to achieve control of MetS were put under investigation by Mahadzir et al. [29, 30] and Suwankruhasn et al. [40]. The former involved 48 Malaysians in a feasibility trial with a pretest-posttest design. The participants underwent weekly peer support sessions led by trained peer leaders for three months and were subsequently followed up for the same period. Assessments were performed by a nutritionist for changes in nutrition and lifestyle behaviors as well as anthropometric measures and metabolic biomarkers [29, 30]. By contrast, the RCT by Suwankruhasn et al. described a support program centered on a self-management strategy designed to positively influence diet and physical activity by improving knowledge, skills, and confidence [40]. Six two-hour sessions involving education, self-management skill training, and discussion were carried out over three months for the intervention group, while the control received standard health education by registered nurses at the hospital diabetes/hypertension clinic [40].

### Metabolic syndrome outcomes

**Fig 3:**
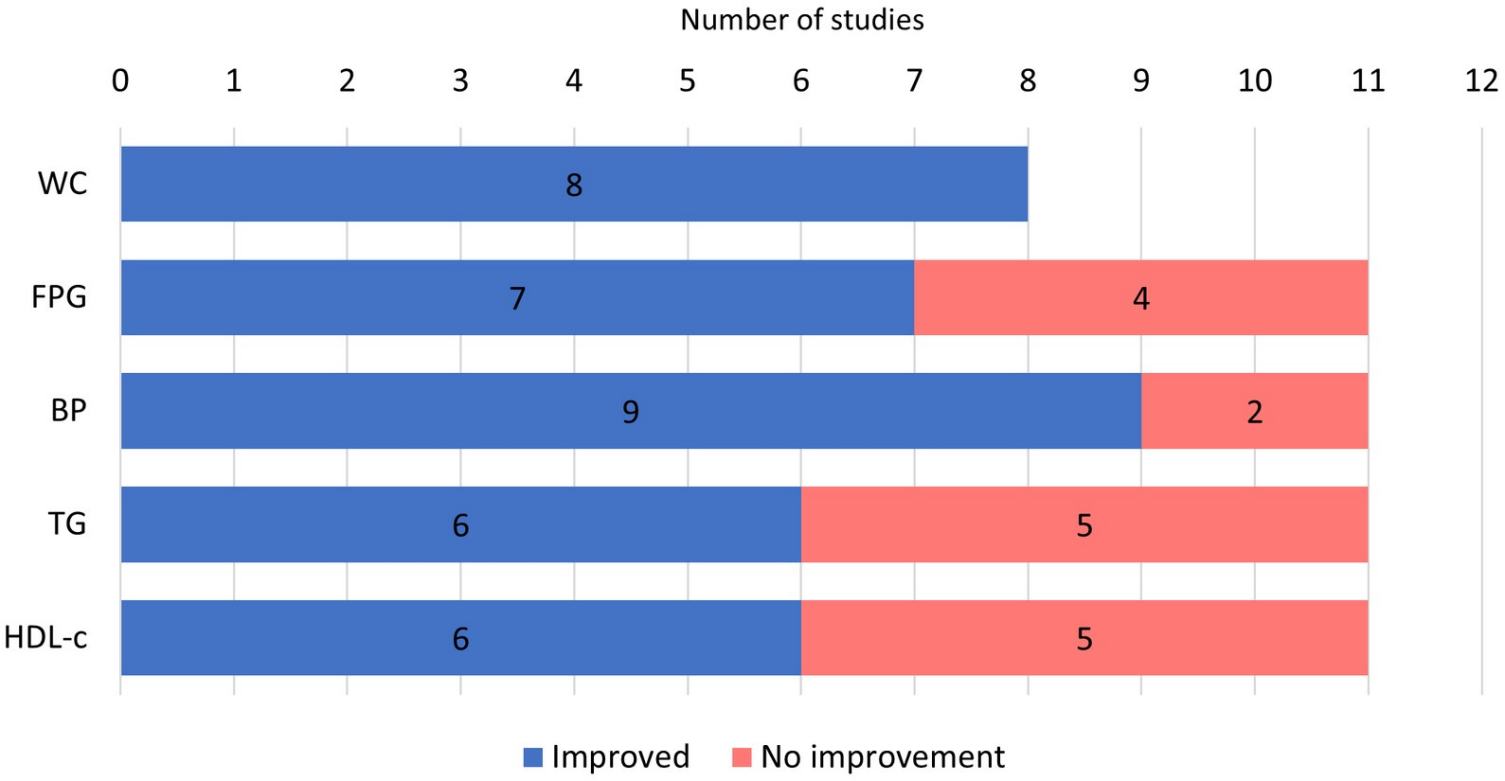
Outcomes for metabolic syndrome components and the proportion presenting statistically significant and non-significant results. WC = waist circumference; FBG = fasting blood glucose; BP = blood pressure; TG = triglyceride; HDL-c = high-density lipoprotein cholesterol.

The number of studies reporting each component of MetS and their proportions which recorded significant improvements compared to either baseline or control is shown in Fig 3. Briefly, all studies that reported waist circumference found significant improvement at post-intervention [27, 30, 32, 33, 36, 37, 39, 40]. More than 80% of studies found significant improvement in blood pressure [27, 30–33, 36, 37, 39, 40], followed by 64% of studies that reported fasting blood glucose [30–32, 36, 37, 39, 40]. Relatively fewer interventions found success in the improvement of lipid profile.

#### Anthropometry and physical measures

Anthropometry and physical measures assessed in the included interventions are presented in Table 2. Waist circumference can be considered a mainstay of abdominal obesity measurement recognized by most definitions of MetS, chiefly the IDF definition, which maintains it as a prerequisite [10]. Eight studies investigated this, and remarkably, all have yielded significant reductions ranging from 0.71% to 4.71% post-intervention [27, 30, 32, 33, 36, 37, 39, 40]. Hip circumference was also measured in two studies [27, 36], but only one produced substantial results [36]. Nevertheless, this was used to calculate the waist-to-hip ratio with significant improvements of about 2.3% in both studies [27, 36]. Used as an anthropometric measure in the WHO [5] and AACE [7] criteria, body mass index (BMI) was reported by five studies [27, 30, 33, 34, 36]. Four of these [27, 30, 33, 36] demonstrated positive outcomes with the largest decline demonstrated in the 2014 physical activity intervention by Chee et al. at 7.36% compared to baseline [36].

**Table 2:** Changes in anthropometry and physical measures.

Measure	Total studies *N*	Studies reporting significant changes *n* (%)	Minimum change^a^ (%)	Maximum change^a^ (%)	References
BMI	5	4 (80.0)	1.00	7.36	[27–30, 33, 34, 36]
Body weight	5	5 (100.0)	0.96	7.27	[27, 28, 31, 33, 36, 39]
WC	8	8 (100.0)	0.71	4.71	[27–30, 32, 33, 36, 37, 39, 40]
HC	2	1 (50.0)	0.13	2.39	[27, 28, 36]
WHR	2	2 (100.0)	2.22	2.30	[27, 28, 36]
Body fat	2	1 (50.0)	1.54	17.10	[29, 30, 36]
Fat mass	2	2 (100.0)	4.15	22.69	[36, 39]
Fat-free mass	1	1 (100.0)	0.44	n/a	[39]
SBP	11	8 (72.7)	2.97	18.30	[27–37, 39, 40]
DBP	11	7 (63.6)	0.95	14.96	[27–37, 39, 40]
Pulse rate	1	0 (0.0)	2.60	n/a	[39]

BMI, body mass index; WC waist circumference; HC, hip circumference; WHR, waist-hip ratio; SBP, systolic blood pressure; DBP, diastolic blood pressure

^a^minimum and maximum percentage change = (change in measure between pre-post intervention / measure at baseline) * 100%

All studies undertook systolic (SBP) and diastolic blood pressure (DBP) analysis [27, 30–37, 39, 40], with a marked reduction observed in eight [27, 30–32, 36, 37, 39, 40] and seven studies [31–33, 36, 37, 39, 40], respectively. The multilevel intervention by Zahtamal et al. recorded the highest drop post-intervention, reducing SBP and DBP by as much as 18.30% and 14.96%, respectively [32]. Although not a component of MetS, body composition measures such as fat and fat-free mass as well as body fat percentage were included in three studies with two exhibiting significant improvements in this respect [36, 39].

#### Glucose and glucose-related parameters

Assessments pertaining to blood glucose and its metabolism (Table 3) play a critical role in the diagnosis of MetS, especially according to the WHO [5], EGIR [6] and AACE [7] criteria. Fasting plasma glucose (FPG) levels were reported by all studies, of which approximately two thirds [30–32, 36, 37, 39, 40] successfully lowered this significantly with a maximum change of 19.17% in the 2017 study by Chee et al. [37]. Interestingly, the community-based behavioral intervention by Tran et al. saw an increase in FPG for both intervention and control groups, but the rise was smaller in the former [27]. Two studies [35, 39] also evaluated fasting serum insulin levels and combined the two measures to compute the Homeostatic Model Assessment of Insulin Resistance (HOMA-IR) score, but only one [39] yielded statistically significant improvement of 20.8% post-intervention.

**Table 3:** Changes in glycemic measures.

Measure	Total studies *N*	Studies reporting significant changes *n* (%)	Minimum change^a^ (%)	Maximum change^a^ (%)	References
FPG	11	7 (63.6)	0.6	19.2	[27–37, 39, 40]
2-hour blood glucose	1	0 (0.0)	19.1	n/a	[31]
Fasting serum insulin	2	1 (50.0)	2.4	20.8	[35, 39]
HbA1c	1	1 (100.0)	3.2	n/a	[39]
HOMA-IR	2	1 (50.0)	0.6	30.4	[35, 39]

FPG, fasting plasma glucose; HbA1c, glycosylated hemoglobin; HOMA-IR, Homeostatic Model Assessment of Insulin Resistance

^a^minimum and maximum percentage change = (change in measure between pre-post intervention / measure at baseline) * 100%

#### Lipid profile

The effect of interventions on two important parameters universally categorized as separate constituents of MetS—serum triglyceride and high-density lipoprotein (HDL)-cholesterol levels—were examined in all studies (Table 4). Six [30, 31, 33, 36, 37, 40] showed significantly reduced serum triglyceride levels, the greatest of which was 42.47% in the aerobics arm of the 2017 study by Chee et al. [37]. Five studies [27, 30, 36, 37, 40] increased HDL-cholesterol concentrations with intervention by up to 77.9%, as seen in Tran et al.’s lifestyle intervention [27].

**Table 4:** Changes in lipid profile.

Measure	Total studies *N*	Studies reporting significant changes *n* (%)	Minimum change^a^ (%)	Maximum change^a^ (%)	References
TG	11	6 (54.5)	0.85	42.5	[27–37, 39, 40]
TC	7	4 (57.1)	0	19.1	[27, 28, 31, 33–36, 39]
HDL-c	11	5 (45.5)	0	77.9	[27–37, 39, 40]
LDL-c	6	3 (50.0)	0.72	21.4	[27, 28, 31, 33, 35, 36, 39]
TG	11	6 (54.5)	0.85	42.5	[27–37, 39, 40]

HDL-c, high-density lipoprotein cholesterol; LDL-c, low-density lipoprotein cholesterol; TC, total cholesterol; TG, triglycerides

^a^minimum and maximum percentage change = (change in measure between pre-post intervention / measure at baseline) * 100%

Other lipid profile measurements included total cholesterol and low-density lipoprotein (LDL)-cholesterol levels, which were investigated by seven and six studies, respectively. Four showed a significant decline in total cholesterol [27, 31, 33, 36], while LDL-cholesterol decreased in three studies [31, 33, 36], with a maximum decline of around 20% for both parameters.

### Nutrition, physical activity and lifestyle outcomes

Given that most studies aimed to improve health through nutritional, physical activity and lifestyle changes, it is important to consider the impact of interventions apart from clinical outcomes (Table 5). Intake of various macronutrients was measured in three studies [30, 32, 40], but only the peer support program by Mahadzir et al. [30] demonstrated significant improvements post-intervention with the largest effect seen in fat consumption (-24.6%). The same studies investigated dietary fiber intake, of which two reported a marked increase [30, 32].

**Table 5:** Changes in nutrient intake, physical activity, and lifestyle measures.

Measure	Total studies *N*	Studies reporting significant changes *n* (%)	Minimum change^a^ (%)	Maximum change^a^ (%)	References
Carbohydrate intake	3	1 (33.3)	0.1	6.7	[29, 30, 32, 40]
Protein intake	3	1 (33.3)	1.3	3.9	[29, 30, 32, 40]
Fat intake	3	1 (33.3)	2.2	24.6	[29, 30, 32, 40]
Fiber intake	3	2 (66.7)	3.6	3.9	[29, 30, 32, 40]
Physical activity level	4	3 (75.0)	8.9	80.3	[27–30, 32, 40]
Step count	3	3 (100.0)	10.6	84.5	[27, 28, 36, 37]
Smoking	1	1 (100.0)	74.9	n/a	[29, 30]
Sleep	1	1 (100.0)	54.8	n/a	[29, 30]

^a^minimum and maximum percentage change = (change in measure between pre-post intervention / measure at baseline) * 100%

Four studies [28, 30, 32, 40] reported on total physical activity and all except one [32] produced significant improvements, with the greatest being over 80% increase from baseline [28]. A more specific measure of physical activity in the form of step count was undertaken by three studies [28, 36, 37] and all showed significant increase of 10.6% to 84.5% with intervention. Sleep and smoking habits were reported by only one study [30] and improvements were observed in both aspects.

## Discussion

To our knowledge, this is the first scoping review conducted to collate and analyze MetS interventions explicitly performed on the population of Southeast Asia and their outcomes both directly and indirectly associated with MetS. Only a handful of studies were identified and deemed appropriate for inclusion in our review, highlighting the dearth of recent interventional endeavors for MetS in the region. The four countries included were nations with relatively high research output in Southeast Asia, as evidenced by a report by the British Council [41] and in accordance with regional findings from another review [42].

Based on the various criteria for MetS put forward by different professional bodies over three decades, it is unsurprising that three MetS definitions were employed collectively by the included studies. However, given that the modified NCEP ATP III and Harmonized criteria bear a striking resemblance in all clinical measures, it is reasonable to assume both as a single definition [43]. Moy and Bulgiba found evidence supporting the use of the modified NCEP ATP III criteria instead of the IDF criteria, particularly among Malays in Malaysia [44]. Most studies in this review were conducted among middle-aged to elderly participants, as they are more susceptible to MetS than those in younger age groups [45, 46]. However, an Australian survey discovered that younger people with MetS were more prone to cardiovascular complications than the aged population [47]. This, together with the increasing prevalence of MetS among youths [48], underscore the need to introduce preventative and therapeutic interventions to combat MetS at an earlier age.

Drugs, supplements, and complementary medicine comprised about 40% of interventions discussed, but they were not representative of the best options available in their respective therapeutic categories. For instance, metformin is among the drug options that may be prescribed in addition to statins, the recommended first-line therapy for MetS patients at a high 10-year risk of cardiovascular disease [49]. Other examples revolve around alternative medicine used by a sizable proportion of Southeast Asians, especially Malaysians and Singaporeans [50]. Several traditional herbs, such as cinnamon, curcumin, and berberine, were commonly reported to exhibit medicinal qualities against MetS [51–53]. Evidence for *Nigella sativa* and *Hibiscus sabdariffa*—medicinal plants from which the two herbal interventions in our review were derived—is also strong with *in vivo* experiments demonstrating generally positive effects on MetS [54, 55]. Results for the included study on black seed oil were less promising compared to similar studies [55, 56], but it is possible that the shorter intervention duration has limited its potential to impart meaningful clinical improvements.

Approximately half of the interventions focused mainly on lifestyle and behavioral improvements through education and support for self-inspired changes, rather than experimental investigations involving the assignment of active interventions. Benefits of a healthy lifestyle can be observed in the United States Diabetic Prevention Program (DPP), which set specific goals for participants to achieve (weight loss of at least 7% and moderate physical activity of at least 150 minutes per week) [57]. This landmark trial successfully reduced diabetes incidence by 58% [57], with secondary analysis demonstrating a 41% decrease in MetS incidence [58]. Many studies have trialed similar strategies based on patient-motivated lifestyle modification in the past with generally favorable outcomes [21, 59]. Such interventions need not be resource-intensive; rather their design should conform to the local cultural context to achieve better health outcomes [60].

In our outcome analysis, we prioritized MetS components while considering particular variables explored by specific studies that may have relevance to MetS, such as nutritional intake, physical activity levels, biomolecular markers, and risk of coronary heart disease. These heterogeneous parameters can be interpreted as risk factors, pathophysiologic elements or complications of MetS. However, not all outcomes showed similar improvements with intervention. Lipid profile measurements were statistically more resistant to change compared with other core MetS components. Studies have found that high triglyceride and low HDL-cholesterol levels are strong predictors of atherosclerosis and cardiovascular risk [61, 62], and aberrant correlations of these parameters were associated with MetS risks even in healthy individuals [63]. Further research should investigate the causes of the relatively modest outcomes for lipid profile, given its critical role in MetS and implications for reducing morbidity and mortality.

Based on the evidence gathered, we propose several recommendations for future MetS interventions in Southeast Asia. Our review only included four countries in the region; thus more studies are required to ascertain the relevance and practicality of MetS interventions in the diverse populations living in various settings in all Southeast Asian countries. The proportion of subjects with MetS before and after the intervention was only formally assessed in one study [27], while others elaborated individual MetS components. Greater focus should be given to this impactful and straightforward measure of intervention performance and effectiveness. Interventions in our review covered specific nutraceuticals and dietary supplements, but not broader interventions on dietary patterns. Examples include low glycemic index foods, high protein diets or even specific dietary styles such as the Mediterranean diet, as Western studies have done in the past [64].

Our scoping review possesses a few strengths, largely in ensuring recent evidence is presented for the perusal of researchers and medical practitioners alike. We have made every effort to incorporate relevant articles by not imposing limitations on the types of interventions and definitions of MetS and performing manual hand-searching in bibliographies of retrieved articles. This review accepts only human interventional study, assuring high quality of evidence for application in clinical practice. Two reviewers were involved in screening and selecting studies for inclusion to minimize selection bias, with a third reviewer confirming the final list of articles and overseeing the process. The review process is rigorously detailed in the Methods section to ensure the transparency and clarity of the scoping review.

On the other hand, a few limitations to our review can be identified. Scoping reviews generally involve a broad range of sources, not limited to online databases, grey literature, electronic search engines, data archives and written scientific texts. We have performed major database searches, which may reduce the number of studies under consideration. The scoping review framework utilized for the review [22, 23] recommended electronic databases and manual-handsearching of reference lists, which has been performed extensively in our review. As noted by Levac et al. [24], while the breadth and depth of search are important, it is not practical to search all possible avenues. This review only accepts studies written in English, allowing a possibility of language bias. However, as journals published in languages other than English are not common in this region, we believe this would not have significant impact on our search results, if at all. Also, the number of articles included is relatively small, with a modest total sample size. The included studies were also not subjected to critical appraisal; hence the quality of individual studies is not known. Most studies provided limited information on long-term outcomes. Importantly, the effects of maintaining lifestyle interventions for extended periods beyond six months could not be observed.

## Conclusion

Modernization in Southeast Asia has brought about increased prevalence of MetS in the region. Various interventions to manage MetS among Southeast Asians have been attempted, largely focused on nutritional supplements and remedies, physical activity and behavioral change through education and group support. Improvements of MetS-related parameters ranged from modest in lipid profile to excellent in anthropometric measures. Future research should inquire into the causes of the underperforming outcomes and report on proportion of subjects with MetS before and after intervention. Emphasis should also be placed on the effects of dietary interventions and the inclusion of the populations of other countries in Southeast Asia.

## Supporting information

S1 Checklist PRISMA Sc-R checklist. (PDF)

S1 Table Definitions of metabolic syndrome. (DOCX)

S2 Table Search strategy in Ovid MEDLINE. (DOCX)

S3 Table Inclusion and exclusion criteria. (DOCX)

S4 Table Summary of included interventions. (DOCX)
