# Supplementary material for: Nutritional and lifestyle intervention strategies for metabolic syndrome in Southeast Asia: A scoping review of recent evidence
Source: PLoS One. 2021 Sep 14;16(9):e0257433. doi: 10.1371/journal.pone.0257433 (PMC8439470; doi:10.1371/journal.pone.0257433)
Supplement: S1 Table — (DOCX) [file pone.0257433.s002.docx]

**S1 Table**. Definitions of metabolic syndrome

| **Component** | **WHO (1998)** | **EGIR (1999)** | **NCEP ATP III (2001)** | **AACE (2003)** | **IDF (2005)** | **Modified NCEP ATP III (2005)** | **Harmonized (2009)** |
| --- | --- | --- | --- | --- | --- | --- | --- |
| Metabolic syndrome diagnosis | T2DM or IFG or IGT  *plus ≥2 of the following* | Fasting insulin >75^th^ percentile  *plus ≥2 of the following* | *≥3 of the following* | IGT or IFG  *plus ≥2 of the following* | Central obesity  *plus ≥2 of the following* | *≥3 of the following* | *≥3 of the following* |
| Anthropometry | WHR >0.9 in men  >0.85 in women  or BMI >30 kg/m^2^ | WC ≥94 cm in men  WC ≥80 cm in women | *For Caucasians:*  WC ≥102 cm in men  WC ≥88 cm in women | BMI >25 kg/m^2^ | *For South Asians:*  WC ≥90 cm in men  WC ≥80 cm in women | *For Asians:*  WC ≥90 cm in men  WC ≥80 cm in women | *For South Asians:*  WC ≥90 cm in men  WC ≥80 cm in women |
| Blood glucose | T2DM or IFG or IGT | IGT or IFG | ≥6.1 mmol/L or T2DM | IGT or IFG | ≥5.6 mmol/L or T2DM | ≥5.6 mmol/L or on specific treatment | ≥5.6 mmol/L or on specific treatment |
| Blood pressure | ≥140/90 mmHg | ≥140/90 mmHg or on hypertension treatment | ≥130/85 mmHg | ≥130/85 mmHg | ≥130/85 mmHg or on hypertension treatment | ≥130/85 mmHg or on hypertension treatment | ≥130/85 mmHg or on hypertension treatment |
| Triglycerides | ≥1.7 mmol/L | ≥1.7 mmol/L | ≥1.7 mmol/L | ≥1.7 mmol/L | ≥1.7 mmol/L or on specific treatment | ≥1.7 mmol/L or on specific treatment | ≥1.7 mmol/L or on specific treatment |
| HDL cholesterol | <1.03 mmol/L in men  <1.29 mmol/L in women | <1.01 mmol/L in men or women | <1.03 mmol/L in men  <1.29 mmol/L in women | <1.03 mmol/L in men  <1.29 mmol/L in women | <1.03 mmol/L in men  <1.29 mmol/L in women  or on specific treatment | <1.03 mmol/L in men  <1.29 mmol/L in women  or on specific treatment | <1.0 mmol/L in men  <1.3 mmol/L in women  or on specific treatment |
| Other | Microalbuminuria | - | - | - | - | - | - |

BMI, body mass index; HDL, high-density lipoprotein; IFG, impaired fasting glucose; IGT, impaired glucose tolerance; T2DM, type 2 diabetes mellitus; WC, waist circumference; WHR, waist-to-hip ratio. Other abbreviations were described in the text.
