# Supplementary material for: Nutritional and lifestyle intervention strategies for metabolic syndrome in Southeast Asia: A scoping review of recent evidence
Source: PLoS One. 2021 Sep 14;16(9):e0257433. doi: 10.1371/journal.pone.0257433 (PMC8439470; doi:10.1371/journal.pone.0257433)
Supplement: S2 Table — (DOCX) [file pone.0257433.s003.docx]

**S2 Table.** Search strategy in Ovid MEDLINE

| **No** | **Query** | **Results** |
| --- | --- | --- |
| 1 | Malaysia/ or Malaysian.mp. | 16814 |
| 2 | Singapore/ or Singaporean.mp. | 13956 |
| 3 | Thailand.mp. or Thailand/ | 33896 |
| 4 | Thai.mp. | 12947 |
| 5 | Indonesia/ or Indonesian.mp. | 11949 |
| 6 | Brunei/ or Bruneian.mp. | 240 |
| 7 | Cambodia.mp. or Cambodia/ | 4448 |
| 8 | Cambodian.mp. | 1427 |
| 9 | Vietnam.mp. or Vietnam/ | 17075 |
| 10 | Vietnamese.mp. | 4861 |
| 11 | Burma.mp. or Myanmar/ | 2988 |
| 12 | Burmese.mp. | 803 |
| 13 | Laotian.mp. or Laos/ | 2103 |
| 14 | Philippines/ or Filipino.mp. | 9491 |
| 15 | Asia, Southeastern/ or Southeast Asian.mp. | 10323 |
| 16 | 1 or 2 or 3 or 4 or 5 or 6 or 7 or 8 or 9 or 10 or 11 or 12 or 13 or 14 or 15 | 116425 |
| 17 | metabolic syndrome.mp. or Metabolic Syndrome/ | 50085 |
| 18 | 16 and 17 | 322 |
| 19 | limit 18 to english language | 321 |
| 20 | limit 19 to "all adult (19 plus years)" | 269 |
| 21 | limit 20 to yr="2010 - 2020" | 208 |
| ^a^ A similar strategy was used with other databases  ^b^ as of 17 February 2021 | | |
