# Supplementary material for: Nutritional and lifestyle intervention strategies for metabolic syndrome in Southeast Asia: A scoping review of recent evidence
Source: PLoS One. 2021 Sep 14;16(9):e0257433. doi: 10.1371/journal.pone.0257433 (PMC8439470; doi:10.1371/journal.pone.0257433)
Supplement: S3 Table — (DOCX) [file pone.0257433.s004.docx]

**S3 Table.** Inclusion and exclusion criteria for this scoping review

| Inclusion criteria |
| --- |
| - Conducted in Southeast Asia - Adult population (≥18 years old) - Published between 1 January 2010 and 31 December 2020 |
|  |
| Exclusion criteria |
| - Wrong study design (i.e. review, meta-analysis, laboratory/basic science research) - Not peer-review full paper (i.e. book, book chapter, abstract, editorial) - Trial protocols |
| - Not MetS-related topic, intervention or outcome - Not an interventional study - Not human study - Performed on children and adolescents - Not Southeast Asian population - Conducted among Southeast Asians living in non-Southeast Asian countries - Featured insufficient components to satisfy any complete formal definitions of MetS - Conducted among healthy participants, or a mix of those with and without MetS but less than 80% had MetS |

MetS; metabolic syndrome
