# Supplementary material for: Nutritional and lifestyle intervention strategies for metabolic syndrome in Southeast Asia: A scoping review of recent evidence
Source: PLoS One. 2021 Sep 14;16(9):e0257433. doi: 10.1371/journal.pone.0257433 (PMC8439470; doi:10.1371/journal.pone.0257433)
Supplement: S4 Table — (DOCX) [file pone.0257433.s005.docx]

**S4 Table.** Summary of included interventions (*n*=11)

| **Study / Country** | **Focus / Study type** | **Samples / MetS criteria** | **Duration of intervention (weeks)** | **Intervention / intervention provider** | **Outcome measures** | **Main findings** |
| --- | --- | --- | --- | --- | --- | --- |
| Chaiyasoot et al (2018)  Thailand | Nutrition & physical activity  RCT | 110 obese adults (≥18 years old)  IDF criteria | 12 | Lifestyle education intervention (LEI) comprising of nutrition and physical activity-related behavioural changes + high-protein meal replacement (MR) vs LEI alone.  Dietitian | Bodyweight, WC, SBP, DBP, pulse rate, FPG, TC, TG, HDL-c, LDL-c, HbA1c, fasting plasma insulin, urine microalbumin and body composition | Significant weight loss was evident in both groups but the effect was greater (p<0.05) in LEI + MR than LEI.  Waist circumference and blood pressure improved significantly in both groups than baseline, but FPG only improved significantly in LEI + MR.  Participants in the LEI + MR group had significantly lower HbA1c, fasting insulin and HOMA-IR than those receiving LEI alone. |
| Chee et al (2014)  Malaysia | Physical activity  RCT | 140 government employees (18-59 years old)  Harmonized criteria | 16 | Physical activity intervention delivered through Facebook vs control (fortnightly group meetings to measure progress).  Dietitian | Anthropometric assessment (weight, BMI, waist circumference, WHR, SBP, DBP), body composition (fat mass, body fat percentage), blood parameters (TC, HDL-c, LDL-c, TG, FPG), and step count. | A significant difference in the number of steps per day between the baseline and the first phase (p<0.001) was observed in both the Facebook and control groups, with a significantly greater increase in the former.  Reduction of metabolic syndrome of 94.3% in the Facebook group after the 4-month intervention, compared to 21.2% for the control group. |
| Chee et al (2017)  Malaysia | Physical activity  RCT | 189 government employees (18-59 years old)  Harmonized criteria | 16 | Point-of-decision (POD) prompt (standing banners and fortnightly group meetings) vs aerobics group (weekly aerobics classes and fortnightly group meetings) vs control group.  Dietitian | Total daily energy expenditure, step counts, waist circumference, blood pressure, HDL-c, triglycerides and blood glucose | Significant time-by-group interaction effect (p<0.001) on step count, with a significantly greater increased observed in the aerobics group than the control group.   Although the POD group significantly improved their step counts compared to baseline, the step counts were not significantly different between the POD group and the control group over time.  The aerobics group experienced the greatest reductions in the proportions of individuals with metabolic syndrome with a post-intervention reduction of 79.4% compared to baseline. |
| Mahadzir et al (2020); Mahadzir et al (2020)  Malaysia | Nutrition and lifestyle behavior  Pre-post feasibility trial | 48 Malaysian adults with MetS  Harmonized criteria | 12 | 2-hour introduction and peer support session, followed by weekly hour-long small-group peer sessions designed to improve nutrition and lifestyle behavior.  Nutritionist | Changes in nutrient intake, dietary behaviour, lifestyle behaviour (physical activity, smoking, and duration of sleep), anthropometry, and metabolic parameters (SBP, DBP, FPG, BMI, WC, BF, TG, HDL-c) | Although total carbohydrate intake and glycemic load increased, total energy and fat intake were significantly reduced post-intervention (all p<0.001)  Compared to baseline, more participants took longer than 20 minutes to eat their main meals (p=0.001) and less skipped breakfast and took supplements (both p<0.001) at post-intervention. The proportion of late-night eaters decreased at follow-up (p=0.039).  The number of physically active peers increased significantly (p<0.001) while the proportion of smokers declined (p=0.031) post-intervention. All participants reportedly had at least 6 hours of sleep per day after intervention, compared to approximately two-thirds at baseline.  Significant improvements were found in all anthropometry and metabolic parameters post-intervention, except for DBP and BF. These changes were not sustained at follow-up. |
| Shahar et al (2013)  Malaysia | Nutrition education  Quasi-experimental design | 47 older Malays diagnosed with MetS (60-75 years old)  IDF criteria | 24 | Nutrition education (4 sessions of group counselling sessions, talks, and cooking and exercise demonstrations using specifically developed healthy aging packages) vs control (general health education package).  Dietitian | Anthropometric and clinical parameters (SBP, DBP, FPG, fasting serum lipids, CRP, body weight, waist circumference), biochemical profile (TC, HDL-c, LDL-c, TG) | Women in the nutrition education group showed a significant reduction in waist circumference (p<0.01) and body weight (p<0.05) compared to the control group. Significant time effects were evident for LDL-c (p<0.05), TG (p<0.01) and DBP (p<0.01).  Men who received nutrition education maintained a stable TC level compared to the increase seen in the control group (p<0.05). |
| Suwankruhasn et al (2013)  Thailand | Self-management Support Program  RCT | 86 MetS patients (50-75 years old)  Modified NCEP ATP III criteria | 12 | Self-management support program (6 sessions involving education, self-management skill training, and discussion) vs control (standard care).  Nurse | Eating behavior (macro-and micronutrient intake), physical activity, waist circumference, SBP, DBP, FPG, TG, HDL-c | Improved physical activity levels at 3 and 6 months, as well as FPG and HDL-c at 6 months in the intervention group. |
| Tran et al (2017); Tran et al (2017)  Vietnam | Nutrition & physical activity  RCT | 417 volunteers with MetS (50-65 years old)  Modified NCEP ATP III criteria | 24 | Community-based physical activity and nutrition intervention programme (four education sessions, an information booklet, a resistance band, and a walking group) vs control (standard diet and physical activity advice).  Public health expert | The proportion of participants with MetS**,** blood parameters (FPG, TC, TG, HDL-c, LDL-c), blood pressure, and anthropometry (waist and hip circumference, WHR, weight, BMI), physical activity, sedentary behavior, dietary behavior (fruit and vegetable intake, intake of animal innards, use of cooking oil and salt) | Significant improvements in HDL-c, waist circumference, WHR, weight, and BMI (all p<0.001) in intervention group.  Significant reduction in the mean number of MetS components only in the intervention group (p<0.001). The proportion of MetS participants was reduced in both intervention and control groups (p<0.001). Comparing between the groups, this was significantly lower in the intervention group (p<0.001).  Significant increases in moderate-intensity activity (p=0.018), walking time (p<0.001), and total physical activity (p=0.001), as well as a reduction in mean sitting time (p<0.001) in the intervention group compared to controls.   The intervention group showed significant reductions in intake of animal internal organs (p=0.001) and the use of cooking oil (p=0.001) compared to the control group over the 6-month period. |
| Wahid et al (2020)  Indonesia | Supplement (black seed oil)  RCT | 62 patients at risk of MetS (>18 years old)  Other MetS criteria | 20 days | Black seed oil vs placebo.  Not known | BMI, SBP, DBP, blood glucose, TG, TC, HDL-c, nuclear factor erythroid 2-related factor 2 (Nrf2) levels | No significant difference in both groups for mean BMI, blood serum glucose, blood pressure (systolic and diastolic), cholesterol levels and Nrf2 levers (p>0.05). |
| Wongwiwatthananukit et al (2013)  Thailand | Vitamin D2 supplementation  RCT | 90 MetS patients >20 years old with vitamin D deficiency (≤20 ng/mL)  Modified NCEP ATP III criteria | 8 | Vitamin D2 40,000 IU per week vs vitamin D2 20,000 IU per week vs placebo  Not known | Serum 25-hydroxyvitamin D [25(OH)D] concentrations, BMI, waist circumference, blood pressure, glycemic (FPG, fasting insulin, HOMA-IR) and lipid profiles (TC, TG, HDL-c, LDL-c), adverse events | Serum 25(OH)D concentrations increased significantly in both vitamin D2 groups when compared with the placebo group (both p<0.001), and were significantly different in both vitamin D2 groups (p=0.040).  The rate of adverse events was not significantly different among the three groups. |
| Yusni et al  (2020)  Indonesia | Supplement (Rosella)  Quasi-experimental  design | 18 elderly women with MetS (>60 years old)  MetS criteria not specified | 3 | Rosella tea (2 grams/5 calyces twice daily after meals) vs control group.  Not known | Bodyweight, SBP, DBP, FPG, postprandial glucose, cholesterol levels, TG, HDL, LDL, NO, serum cortisol | Bodyweight, SBP, DBP, FPG, TC, TG, HDL-c, LDL-c, and cortisol levels decreased significantly, whereas NO levels increased significantly (all p<0.05) after rosella consumption. |
| Zahtamal et al (2017)  Indonesia | Multilevel intervention  Quasi-experimental design | 34 employees from two oil refining and plantation companies (24-54 years old)  Harmonized criteria | 12 | Multilevel intervention (health education lectures and printed materials, collaborative learning, personal counseling, social support, and advocacy) vs conventional intervention (health education lectures for workers only)  Public health expert(s) | Nutrient intake (protein, carbohydrate, fat, polyunsaturated fatty acids, monounsaturated fatty acids, saturated fatty acids, fiber, cholesterol), abdominal circumference, SBP, DBP, FPG, HDL-c, TG | The multilevel intervention significantly increased dietary fiber intake (p=0.028) post-intervention, while other changes to nutrient intake were insignificant.  Compared to baseline, DBP and FPG were significantly improved for both groups (p<0.05), but only the multilevel intervention resulted in improved SBP (p=0.002). |

BF, body fat; BMI, body mass index; CRP, C-reactive protein; DBP, diastolic blood pressure; FPG, fasting plasma glucose; HbA1c, glycosylated hemoglobin; HDL-c, high-density lipoprotein cholesterol; HOMA-IR, Homeostatic Model Assessment for Insulin Resistance; IDF, International Diabetes Federation; LDL-c, low-density lipoprotein; MetS, metabolic syndrome; NCEP ATP III, National Cholesterol Education Program Adult Treatment Panel III; NO, nitric oxide; RCT, randomized controlled trial; SBP, systolic blood pressure; TC, total cholesterol; TG, triglycerides; WC, waist circumference; WHR, waist-to-hip ratio
